# Supplementary material for: The large GTPase Sey1/atlastin mediates lipid droplet- and FadL-dependent intracellular fatty acid metabolism of Legionella pneumophila
Source: eLife. 2023 May 9;12:e85142. doi: 10.7554/eLife.85142 (PMC10259473; doi:10.7554/eLife.85142)
Supplement: Supplementary file 2. [file elife-85142-supp2.docx]

**Supplementary File 2. Cells, bacterial strains, and plasmids used in this study.**

| **Strain or plasmid** | **Relevant properties** ^a^ | **Reference** |
| --- | --- | --- |
| ***D. discoideum*** |  |  |
| Ax3 | Parental strain | (Loovers et al., 2007) |
| Δ*sey1* | Ax3, insertion in gene DDB_G0279823, Bls^R^ | (Hüsler et al., 2021) |
| ***E. coli*** |  |  |
| TOP10 |  | Invitrogen |
| ***L. pneumophila*** |  |  |
| ER01 (Δ*legG1*) | JR32 *legG1*::Kan^R^ | (Rothmeier et al., 2013) |
| GS3011 (Δ*icmT*) | JR32 *icmT3011*::Kan^R^ | (Segal and Shuman, 1998) |
| JR32 | Derivative of wild-type *L. pneumophila* strain Philadelphia-1 (serogroup 1) | (Sadosky et al., 1993) |
| PS01 (Δ*fadL*) | JR32 *fadL*::Kan^R^ | This study |
| **Plasmids** |  |  |
| pAW016 | pDM323-*calnexin*-*gfp*, G418^R^, Amp^R^ | (Bärlocher et al., 2017) |
| pBS001 | pDM317-*gfp*-*sey1*, G418^R^, Amp^R^ | (Steiner et al., 2017) |
| pCM009 | pMMB207C-P*_flaA_*-*gfp* (ASV), Cam^R^ | (Schell et al., 2016) |
| pCR077 | pMMB207C-P_tac_-RBS-*dsred*-RBS-MCS, Cam^R^ | (Finsel et al, 2013) |
| pDM317 | *Dictyostelium* expression vector, extra-chromo-somal, N-terminal GFP, G418^R^, Amp^R^ | (Veltman et al., 2009) |
| pDM323 | *Dictyostelium* expression vector, extra-chromo-somal, C-terminal GFP, G418^R^, Amp^R^ | (Veltman et al., 2009) |
| pDM329 | *Dictyostelium* expression/shuttle vector, extra-chromosomal, C-terminal GFP, G418^R^, Amp^R^ | (Veltman et al., 2009) |
| pDM1042 | *Dictyostelium* expression vector, extra-chromo-somal, N-terminal mCherry, Hyg^R^, Amp^R^ | (Barisch et al., 2015) |
| pDM1044 | *Dictyostelium* expression vector, extra-chromo-somal, C-terminal mCherry, Hyg^R^, Amp^R^ | (Barisch et al., 2015) |
| pER005 | pCR077-M45-*legG1*, Cam^R^ | (Rothmeier et al., 2013) |
| pER017 | pDXA-*gfp*-*legG1*, Amp^R^ | (Swart et al, 2020) |
| pHK101 | pDM317-*gfp*-*plin*, G418^R^, Amp^R^ | (Koliwer-Brandl et al., 2019) |
| pHK102 | pDM1042-*mCherry*-*plin*, Hyg^R^, Amp^R^ | (Koliwer-Brandl et al., 2019) |
| pHK121 | pDM323-*amtA*-*gfp,* G418^R^, Amp^R^ | (Koliwer-Brandl et al., 2019) |
| pLAW344 | *oriT* (RK2), *oriR* (ColE1), *sacB*, Cam^R^, Amp^R^ | (Wiater et al., 1994) |
| pLS110 | pDM317-*gfp*-*tubA*, G418^R^, Amp^R^ | (Hüsler et al., 2021) |
| pLS117 | pDM317-*gfp*-*legG1*, G418^R^, Amp^R^ | This study |
| pLS187 | pDM323-*ranBP1-gfp*, G418^R^, Amp^R^ | This study |
| pLS221 | pDM1044-*ranA-mCherry,* Hyg^R^, Amp^R^ | This study |
| pLS222 | pDM329-*ranBP1-gfp*, G418^R^, Amp^R^ | This study |
| pNT28 | pMMB207C, Δ*lacI*^q^, P*_tac_*-*gfp* (const.), Cam^R^ | (Tiaden et al., 2007) |
| pNP99 | pMMB207C, Δ*lacI*^q^, P*_tac_*-*mCerulean* (const.), Cam^R^ | (Steiner et al., 2017) |
| pPS001 | pUC19, *fadL*::Kan^R^ | This study |
| pPS002 | pLAW344, *fadL*::Kan^R^ | This study |
| pPS003 | pMMB207C-P*_fadL_*-*gfp* (ASV), Cam^R^ | This study |
| pPS013 | pLAW344, *fadL*::Kan^R^::*fadL* | This study |
| pSU17 | pDXA-HC-*ranA-gfp*, G418^R^, Amp^R^ | (Rothmeier et al., 2013) |
| pSU26 | pDXA-HC-*ranBP1-gfp*, G418^R^, Amp^R^ | (Rothmeier et al., 2013) |
| pSW001 | pMMB207C, Δ*lacI*^q^ , P*_tac_*-*dsRed* (const.), Cam^R^ | (Mampel et al, 2006) |
| pUC4K | *oriR* (pBR322), Amp^R^, MCS::Kan^R^ | Amersham, UK |
| pUC19 | *oriR* (pBR322), Amp^R^ | (Norrander et al., 1983) |
| pWS032 | pDM1044-P4C*_SidC_*-*mCherry,* Hyg^R^, Amp^R^ | (Steiner et al., 2017) |
| pWS034 | pDM323-*P4C_SidC_*-*gfp,* G418^R^, Amp^R^ | (Welin et al., 2018) |

^a^ Abbreviations: Amp, ampicillin; Bls, blasticidin S; Cam, chloramphenicol; Hyg, hygromycin; Kan, kanamycin; G418, geneticin.

**References**

Barisch, C., Paschke, P., Hagedorn, M., Maniak, M., and Soldati, T. (2015) Lipid droplet dynamics at early stages of *Mycobacterium marinum* infection in *Dictyostelium*. *Cell Microbiol* **17**: 1332-1349.

Bärlocher, K., Hutter, C.A.J., Swart, A.L., Steiner, B., Welin, A., Hohl, M. et al. (2017) Structural insights into *Legionella* RidL-Vps29 retromer subunit interaction reveal displacement of the regulator TBC1D5. *Nat Commun* **8**: 1543.

Finsel, I., Ragaz, C., Hoffmann, C., Harrison, C.F., Weber, S., van Rahden, V.A. et al. (2013) The *Legionella* effector RidL inhibits retrograde trafficking to promote intracellular replication. *Cell Host Microbe* **14**: 38-50.

Hüsler, D., Steiner, B., Welin, A., Striednig, B., Swart, A.L., Molle, V. et al. (2021) *Dictyostelium* lacking the single atlastin homolog Sey1 shows aberrant ER architecture, proteolytic processes and expansion of the *Legionella*-containing vacuole. *Cell Microbiol* **23**: e13318.

Koliwer-Brandl, H., Knobloch, P., Barisch, C., Welin, A., Hanna, N., Soldati, T., and Hilbi, H. (2019) Distinct *Mycobacterium marinum* phosphatases determine pathogen vacuole phosphoinositide pattern, phagosome maturation, and escape to the cytosol. *Cell Microbiol* **21**: e13008.

Loovers, H.M., Kortholt, A., de Groote, H., Whitty, L., Nussbaum, R.L., and van Haastert, P.J. (2007) Regulation of phagocytosis in *Dictyostelium* by the inositol 5-phosphatase OCRL homolog Dd5P4. *Traffic* **8**: 618-628.

Mampel, J., Spirig, T., Weber, S.S., Haagensen, J.A.J., Molin, S., and Hilbi, H. (2006) Planktonic replication is essential for biofilm formation by *Legionella pneumophila* in a complex medium under static and dynamic flow conditions. *Appl Environ Microbiol* **72**: 2885-2895.

Norrander, J., Kempe, T., and Messing, J. (1983) Construction of improved M13 vectors using oligodeoxynucleotide-directed mutagenesis. *Gene* **26**: 101-106.

Rothmeier, E., Pfaffinger, G., Hoffmann, C., Harrison, C.F., Grabmayr, H., Repnik, U. et al. (2013) Activation of Ran GTPase by a *Legionella* effector promotes microtubule polymerization, pathogen vacuole motility and infection. *PLoS Pathog* **9**: e1003598.

Swart, A.L., Steiner, B., Gomez-Valero, L., Schütz, S., Hannemann, M., Janning, P. et al. (2020) Divergent evolution of *Legionella* RCC1 repeat effectors defines the range of Ran GTPase cycle targets. *mBio* **11**: e00405-20.

Sadosky, A.B., Wiater, L.A., and Shuman, H.A. (1993) Identification of *Legionella pneumophila* genes required for growth within and killing of human macrophages. *Infect Immun* **61**: 5361-5373.

Schell, U., Simon, S., Sahr, T., Hager, D., Albers, M.F., Kessler, A. et al. (2016) The alpha-hydroxyketone LAI-1 regulates motility, Lqs-dependent phosphorylation signalling and gene expression of *Legionella pneumophila*. *Mol Microbiol* **99**: 778-793.

Segal, G., and Shuman, H.A. (1998) Intracellular multiplication and human macrophage killing by *Legionella pneumophila* are inhibited by conjugal components of IncQ plasmid RSF1010. *Mol Microbiol* **30**: 197-208.

Steiner, B., Swart, A.L., Welin, A., Weber, S., Personnic, N., Kaech, A. et al. (2017) ER remodeling by the large GTPase atlastin promotes vacuolar growth of *Legionella pneumophila*. *EMBO Rep* **18**: 1817-1836.

Tiaden, A., Spirig, T., Weber, S.S., Brüggemann, H., Bosshard, R., Buchrieser, C., and Hilbi, H. (2007) The *Legionella pneumophila* response regulator LqsR promotes host cell interactions as an element of the virulence regulatory network controlled by RpoS and LetA. *Cell Microbiol* **9**: 2903-2920.

Veltman, D.M., Akar, G., Bosgraaf, L., and Van Haastert, P.J.M. (2009) A new set of small, extrachromosomal expression vectors for *Dictyostelium discoideum*. *Plasmid* **61**: 110-118.

Welin, A., Weber, S., and Hilbi, H. (2018) Quantitative imaging flow cytometry of *Legionella*-infected *Dictyostelium* amoebae reveals the impact of retrograde trafficking on pathogen vacuole composition. *Appl Environ Microbiol* **84**: e00158-00118.

Wiater, L.A., Sadosky, A.B., and Shuman, H.A. (1994) Mutagenesis of *Legionella pneumophila* using Tn*903*dll*lacZ*: identification of a growth-phase-regulated pigmentation gene. *Mol Microbiol* **11**: 641-653.
