# Supplementary material for: The large GTPase Sey1/atlastin mediates lipid droplet- and FadL-dependent intracellular fatty acid metabolism of Legionella pneumophila
Source: eLife. 2023 May 9;12:e85142. doi: 10.7554/eLife.85142 (PMC10259473; doi:10.7554/eLife.85142)
Supplement: Supplementary file 3. [file elife-85142-supp3.docx]

**Supplementary File 3. Oligonucleotides used in this study.**

| **Oligo** | **Sequence (5’ - 3’)** ^a^ | **Comments** |
| --- | --- | --- |
| oLS178 | AAAAAGCGAGATCTAAATTGCATCTTGAATTGCATC | 5’ of *legG1* (forward), *Bgl*II |
| oLS180 | AAAAACGCACTAGTTAACAAATTGCATGGCG | 3’ of *legG1* (reverse), *Spe*I |
| oLS296 | AAAAAGCGAGATCTAAAATGTCAGAAGTCGAAAAGAAAG | 5’ of *ranBP1* (forward), *Bgl*II |
| oLS297 | TTTTTCGCCGCACTAGTTTTTTCAGTTTTTTTTTCAATTGCTTC | 3’ of *ranBP1* (reverse), *Spe*I |
| oLS333 | AAAAAGCGAGATCTAAAATGGCAGAAAAAGAACAAAT | 5’ of *ranA* (forward), *Bgl*II |
| oLS334 | TTTTTCGCCGCACTAGTCAAGTCATCATTGTC | 3’ of *ranA* (reverse), *Spe*I |
| oPS001 | GAGCTCGGTACCCGGGGGATCCTGCTGCTGAAGTATCAG | 5’ of *fadL* 5’ flank (forward), *Bam*HI |
| oPS002 | CGAGGCAGACCTCAGCAGATCTGGTAATAAAAATGATAATAAATGAATTTATA | 3’ of *fadL* 5’ flank (reverse), *Bgl*II |
| oPS003 | TATAAATTCATTTATTATCATTTTTATTACCAGATCTGCTGAGGTCTGCCTCG | 5’ of Kan^R^ (forward), *Bgl*II |
| oPS004 | CAAGGTCAAGAGAAAGCTAGATCTGGGAAAGCCACGTTGTG | 3’ of Kan^R^ (reverse), *Bgl*II |
| oPS005 | CACAACGTGGCTTTCCCAGATCTAGCTTTCTCTTGACCTTG | 5’ of *fadL* 3’ flank (forward), *Bgl*II |
| oPS006 | CAGGTCGACTCTAGAGGATCGGATCCAACAAGCAATGACTGAAAG | 3’ of *fadL* 3’ flank (reverse), *Bam*HI |
| oPS010 | TCCTGCAGCCCGGGGGATCCTGCTGCTGAAG | 5’ of *fadL* 5’ flank (forward), *Bam*HI |
| oPS011 | CGCTCTAGAACTAGTGGATCCAACAAGCAATGACTGAAAG | 3’ of *fadL* 3’ flank (reverse), *Bam*HI |
| oPS013 | GGAAACAGAATTCGAGCTCGGTTAGCCATTGCTG | 5’ of P*_fadL_* (forward), *Sac*I |
| oPS015 | CATATGTATATCTCCTTCTTAAATCTAGAGGTAATAAAAATGATAATAAATG | 3’ of P*_fadL_* (reverse), *Xba*I |
| oPS045 | TCCTGCAGCCCGGGGGATCCTGCTGCTGAAGTATCAG | 5’ of *fadL* 5’ flank (forward), *Bam*HI |

^a^ Restriction sites and regions overlapping with destination vectors or neighbouring assembly fragments are underlined.
